# Host–Pathogen Interaction in Invasive Salmonellosis

**DOI:** 10.1371/journal.ppat.1002933

**Published:** 2012-10-04

**Authors:** Hanna K. de Jong, Chris M. Parry, Tom van der Poll, W. Joost Wiersinga

**Affiliations:** 1 Center for Infection and Immunity Amsterdam (CINIMA), Academic Medical Center, Amsterdam, the Netherlands; 2 Center for Experimental and Molecular Medicine (CEMM), Academic Medical Center, Amsterdam, the Netherlands; 3 Mahidol-Oxford Tropical Medicine Research Unit, Faculty of Tropical Medicine, Mahidol University, Bangkok, Thailand; 4 Centre for Tropical Medicine, Nuffield Department of Clinical Medicine, Churchill Hospital, University of Oxford, Oxford, United Kingdom; 5 Department of Medicine, Division of Infectious Diseases, Academic Medical Center, Amsterdam, the Netherlands; International Centre for Genetic Engineering and Biotechnology, India

## Abstract

*Salmonella enterica* infections result in diverse clinical manifestations. Typhoid fever, caused by *S. enterica* serovar Typhi (*S.* Typhi) and *S.* Paratyphi A, is a bacteremic illness but whose clinical features differ from other Gram-negative bacteremias. Non-typhoidal *Salmonella* (NTS) serovars cause self-limiting diarrhea with occasional secondary bacteremia. Primary NTS bacteremia can occur in the immunocompromised host and infants in sub-Saharan Africa. Recent studies on host–pathogen interactions in Salmonellosis using genome sequencing, murine models, and patient studies have provided new insights. The full genome sequences of numerous *S. enterica* serovars have been determined. The *S.* Typhi genome, compared to that of *S.* Typhimurium, harbors many inactivated or disrupted genes. This can partly explain the different immune responses both serovars induce upon entering their host. Similar genome degradation is also observed in the ST313 *S.* Typhimurium strain implicated in invasive infection in sub-Saharan Africa. Virulence factors, most notably, type III secretion systems, Vi antigen, lipopolysaccharide and other surface polysaccharides, flagella, and various factors essential for the intracellular life cycle of *S. enterica* have been characterized. Genes for these factors are commonly carried on *Salmonella* Pathogenicity Islands (*S*PIs). Plasmids also carry putative virulence-associated genes as well as those responsible for antimicrobial resistance. The interaction of *Salmonella* pathogen-associated molecular patterns (PAMPs) with Toll-like receptors (TLRs) and NOD-like receptors (NLRs) leads to inflammasome formation, activation, and recruitment of neutrophils and macrophages and the production of pro-inflammatory cytokines, most notably interleukin (IL)-6, IL-1β, tumor necrosis factor (TNF)-α, and interferon-gamma (IFN)-γ. The gut microbiome may be an important modulator of this immune response. *S.* Typhimurium usually causes a local intestinal immune response, whereas *S.* Typhi, by preventing neutrophil attraction resulting from activation of TLRs, evades the local response and causes systemic infection. Potential new therapeutic strategies may lead from an increased understanding of infection pathogenesis.

## Introduction

**Figure 1:**
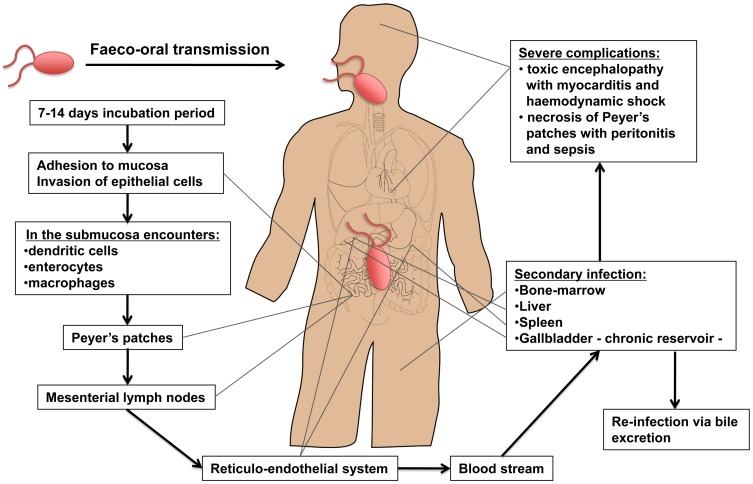
Dissemination of *S.* Typhi during systemic infection. Typhoid is usually contracted by ingestion of food or water contaminated by fecal or urinary carriers excreting *S.* Typhi. The incubation period is usually 7 to 14 d. In the small intestine the bacteria adhere to the mucosa and then invade the epithelial cells. The Peyer's patches, which are aggregrated lymphoid nodules of the terminal ileum, play an important role in the transport to the underlying lymphoid tissue. Specialized epithelial cells such as M cells overlying these Peyer's patches are probably the site of internalization of *S.* Typhi. Once the bacteria have penetrated the mucosal barrier, the invading organism translocates to the intestinal lymphoid follicles and the draining mesenteric lymph nodes, and some pass on to the reticuloendothelial cells of the liver and spleen. During the bacteremic phase, the bacteria are widely disseminated throughout the body. Secondary infection can occur with liver, spleen, bone-marrow, gallbladder, and Peyer's patches as the most preferred sites. The gallbladder is the main reservoir during a chronic infection with *S.* Typhi and invasion occurs either directly from the blood or by retrograde spread from the bile. Of interest, the ability of *Salmonella* to form biofilms on gallstones is likely to be a critical factor in establishment of chronic carriage and shedding of *S.* Typhi [88]. The bacteria that are excreted in the bile can then reinvade the intestinal wall by the mechanism previously described or are excreted by feces. Typical clinical symptoms are fever, malaise, and abdominal discomfort. Clinical features such as a tender abdomen, hepatomegaly, splenomegaly, and a relative bradycardia are common. Rose spots, the classical skin lesions associated with typhoid fever, are relatively uncommon and occur in 5%–30% of cases. The most severe manifestations of typhoid leading to sepsis and death are either necrosis of the Peyer's patches resulting in gut perforation and peritonitis or a toxic encephalopathy associated with myocarditis and haemodynamic shock [8], [89].

Typhoid fever is a global problem, with more than 27 million cases worldwide each year resulting in an estimated 217,000 deaths [1]. *Salmonella enterica* serovar Typhi (*S.* Typhi) and *S.* Paratyphi A are the Gram-negative bacteria that cause this debilitating condition. It is most common among children, especially in areas of Asia and Africa that lack clean water and adequate sanitation, and is also an important travel-associated disease [2]. *S.* Typhi is an exclusively human pathogen causing a bacteremic disease that, unlike many other Gram-negative bacteremias, does not typically manifest with neutrophilia or septic shock [3]. The widespread appearance of antimicrobial-resistant strains has limited treatment options [4], [5]. Relapse and chronic asymptomatic fecal carriage may complicate the illness (Figure 1) [6], [7]. Mortality usually results from intestinal perforation and peritonitis or from a severe toxic encephalopathy associated with myocarditis and hemodynamic shock [8].

Infections with non-typhoidal *Salmonella* (NTS) serovars, such as *S. enterica* serovar Typhimurium and *S.* Enteriditis, also cause a significant disease burden, with an estimated 93.8 million cases worldwide and 155,000 deaths each year (see [9] for review) [10]. NTS serovars usually cause self-limiting diarrhea with secondary bacteremia occurring in less than 10% of patients. The host range of non-typhoidal *Salmonella* serovars is broad, including poultry and cattle, and NTS infection is commonly due to food poisoning in developed countries. NTS serovars cause high rates of bacteremia in the immunocompromised and, in sub-Saharan Africa, in children below 5 years old and those with HIV infection [9], [11]. Antimicrobial resistance is widespread [12].

The variations in the clinical features of infection with this intracellular pathogen relate to differences in the interaction between different *Salmonella* serovars and the host. This review summarizes new and significant insights concerning the virulence factors of both typhoid and non-typhoidal *Salmonellae*, their difference at the genome level, novel mechanisms employed by these intruders to circumvent the host defense, and their interactions with both host microbiome and the innate immune system.

## The Bacteria

### Taxonomy and Genomics of *Salmonella*

The genus *Salmonella* is composed of two distinct species: *Salmonella bongori* and *Salmonella enterica*, the latter being divided into six subspecies. These subspecies are classified into more than 50 serogroups based on the O (somatic) antigen, and divided into >2,400 serovars based on the H (flagellar) antigen. Complete genome sequence from multiple *Salmonella* strains are available [13]. For example, the *S.* Typhi type strain Ty2, the multidrug-resistant (MDR) isolate CT18, and the *S.* Typhimurium strain LT2 are composed of 4.79 (Ty2), 4.86 (CT18), and 4.81 (LT2) megabases, respectively [14]–[16]. The core genomes of *Escherichia coli* and *S. enterica* differ by only 10% in their DNA sequences and suggest that the two species derived from a common ancestor about 100 million years ago. Comparison of different *S.* Typhi isolates show that they are highly related (clonal) and have arisen from a single point of origin approximately 30,000–50,000 years ago [17]. The sequence-based technique of MLST (multilocus sequence typing) provides a more accurate indication of the genomic relationship between different *Salmonella* isolates and may supersede serotyping in the future [18]. Of the ∼4,000 *S.* Typhi genes, more than 200 are functionally disrupted or inactive, while most of these homologs are still fully functional in *S.* Typhimurium. This could in part explain the restricted host range of *S.* Typhi [16]. Although it has been suggested that the different clinical outcomes of infection between typhoid and NTS serovars may be explained by differences in genome expression leading to differences in host-pathogen recognition, one should also consider the opposite possibility; that is, differences in host-pathogen interaction may make certain genes dispensable, resulting in the accumulation of pseudogenes [3], [19], [20].

Recent analysis of *S.* Typhimurium isolates from the unusually invasive infections seen in sub-Saharan Africa have shown dominance of a particular MLST type, ST313, distinct from the usual *S.* Typhimurium sequence type, ST19, associated in other parts of the world with diarrhea. In the D23580 invasive *S.* Typhimurum isolate from Malawi, there is loss of gene function, including genes previously implicated in the virulence of *S.* Typhimurium in the murine model of infection, such as *sseI* (encoding a type III-secreted effector protein) and *ratB* (encoding a secreted protein associated with intestinal persistence), and of the 44 novel pseudogenes or deletions in the strain relative to LT2, 26 are also pseudogenes or deletions in *S.* Typhi or *S.* Paratyphi A [21]. These observations suggest that a similar process of adaption to the human host may be occurring in African *S.* Typhimurium as has been observed in *S.* Typhi.

### Virulence Factors

**Figure 2:**
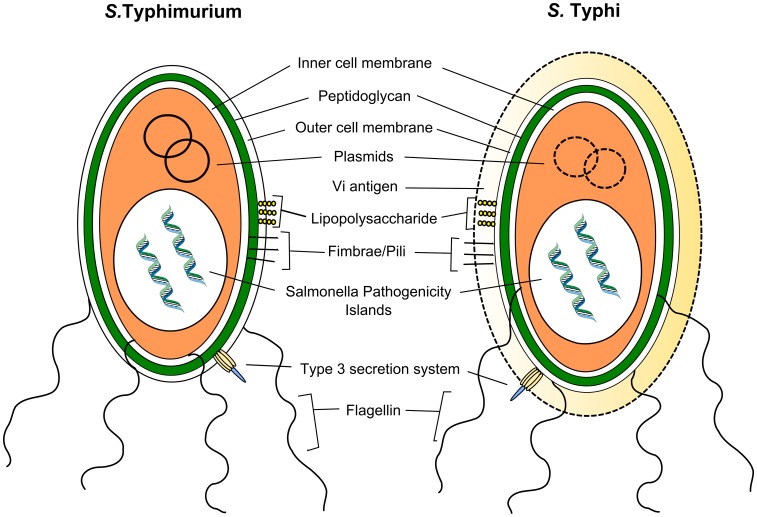
Virulence of *S.* Typhimurium and *S.* Typhi. *S.* Typhimurium and *S.* Typhi possess partly overlapping and a partly distinct repertoire of virulence factors. Both serovars express the type III secretion system, lipopolysaccharide, and other surface polysaccharides, fimbrae, flagellin, and bacterial DNA. The Vi antigen is exclusively expressed by *S.* Typhi and is able to circumvent the innate immune response by repressing flagellin and LPS expression. *S*PI, *Salmonella* pathogenicity islands.

About 90% of the genes in *S.* Typhi and *S.* Typhimurium serovars are identical [16]. The 10% of genes that differ include virulence factors, which determine their pathogenic potential (Figure 2) [20]. The virulence factors of the *Salmonella* serovars are mostly encoded on the *Salmonella* pathogenicity islands (*S*PI).

#### Plasmids and prophages

Integrated bacteriophages, phage remnants, or plasmids are single- or double-stranded DNA molecules that can be exchanged between bacteria by horizontal gene transfer. They give bacteria the opportunity to pass on or receive selected genes that may enhance virulence or result in antimicrobial resistance [17], [22]. Certain *Salmonella* spp. have a self-transmissible virulence plasmid called pSLT, which harbors the *spv* genes. The spvB enzyme, which acts as an intracellular ADP-ribosylating toxin causing host cytotoxicity, is necessary for intra-macrophage survival but is absent in *S.* Typhi and *S.* Paratyphi A [20], [23]. Putative virulence-associated plasmid has only recently been identified in *S.* Typhi. The chimeric plasmid pR(ST98) carries genes that are involved in drug resistance and apoptosis induction in macrophages [24], [25]. Additionally, a linear plasmid in *S.* Typhi strains originating from Indonesia, called pBSSB1, carries the *fljB^z66^* gene, which encodes a flagellin antigen known as H:z66 [26]. Whether the presence of these particular plasmids has an impact on the virulence of *S.* Typhi is not known.

The role of plasmids carrying antimicrobial resistance genes, such as *cat*, *dhfr7*, *dhfr14*, *sul1*, and *bla*_TEM-1_, in the transfer and spread of antimicrobial resistance has been well described [20], [27]. *S.* Typhi is able to exchange multidrug resistance R-plasmids with *E. coli* and other enteric bacteria [28], [29]. The self-transmissible incompatibility group (Inc)HI1 plasmids almost exclusively confer the phenotype of MDR *S.* Typhi. The presence of the MDR phenotype has been suggested to be associated with the development of severe or fatal disease [30], [31]. The presence of a composite genetic element encoding multiple antimicrobial resistance genes on the virulence-associated plasmid in the ST313 serovar Typhimurium isolates causing invasive disease in Africa may provide an explanation for a linkage between drug resistance and an invasive phenotype [21]. Prophages and phage remnants can carry non-essential “cargo” genes involved in bacterial virulence including several type III secretion system effectors, which play an important role in *Salmonella* virulence [20], [32].

#### Type III secretion system and outer membrane vesicles

**Figure 3:**
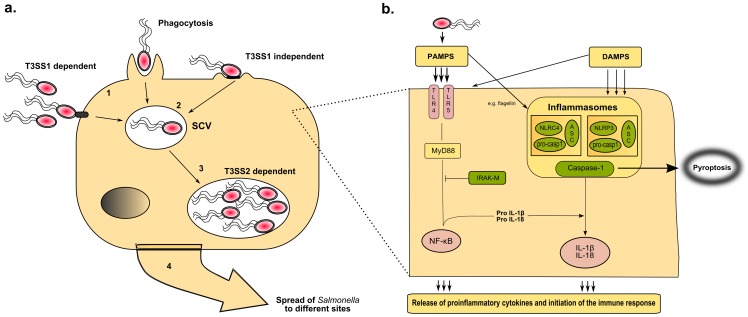
*Salmonella* and its first encounter with the host. (a) The intracellular life of *Salmonella*. Invasion of phagocytic and non-phagocytic cells. *Salmonella* is a facultative intracellular pathogen that can be found in a variety of phagocytic and non-phagocytic cells, in which it is able to survive and replicate. To establish this intracellular niche, the T3SS1 and -2 play a predominant role; key virulence factors are involved in accessing and utilizing these cells [36]. After ingestion, intestinal colonization follows and *Salmonella* enters enterocytes and dendritic cells in the intestinal epithelium [36]. Subsequently, *Salmonella* that reach the submucosa can be internalized by resident macrophages via different mechanisms: by phagocytosis, active invasion using the T3SS1 or T3SS1-independent invasion using fimbriae or other adhesins on the bacterial surface. (1) *Salmonella*-containing-vacuole. Following internalization *Salmonella* remains within a modified phagosome known as the *Salmonella* containing vacuole (SCV) and injects a limited number of effector proteins, such as SipA, SipC, SopB/SigD, SodC-1, SopE2, and SptP into the cytoplasm. These effectors cause rearrangements of the actin cytoskeleton and SCV morphology among other changes. (2) Replication within the SCV. *Salmonella* survives and replicates within the SCV, where it is able to avoid host antimicrobial effector mechanisms. The T3SS2 is required for systemic virulence in the mouse and survival within macrophages. (3) Transport of *Salmonella* to distant sites. After penetration of the M cells, the invading microorganisms translocate to the intestinal lymphoid follicles and the draining mesenteric lymph nodes, and some pass on to the reticuloendothelial cells of the liver and spleen. *Salmonella* organisms are able to survive and multiply within the mononuclear phagocytic cells of the lymphoid follicles, liver, and spleen [36]. (b) Host–pathogen interaction in typhoid and non-typhoid *Salmonella*. Simplified scheme of the first encounter between *Salmonella* spp. and the immune system. Specified cells such as neutrophils, macrophages, dendritic, phagocytic, and epithelial cells recognize specific pathogen associated molecular patterns (PAMPs) and danger-associated-molecular patterns (DAMPs), thereby eliciting an immune response. PAMPs such as LPS, Flagella, and bacterial DNA can trigger TRL4, TRL5, and TRL9, respectively. TLR-induced activation of NF-κB is essential for the production of pro-IL-1β, pro-IL-18, which can be negatively regulated by IRAK-M [90]. The NLRs are situated in the cytosol and can also recognize PAMPs. However, NLRP3 is triggered by a different, yet unknown, mechanism, although DAMPs are thought to play a crucial role. TLR, toll-like receptors; LPS, lipopolysaccharide; NF-κB, regulated nuclear factor kappa-light-chain-enhancer of activated B cells; IRAK-M, IL-1R-assiociated kinase-M; IL, Interleukin; ASC, apoptotic speck protein containing a caspase recruitment domain; NLR, NOD-like receptors (including NLRP3 and NLRC4); MyD88, myeloid differentiation primary response gene [88].

*Salmonella enterica* spp. contains two type III secretion system (T3SS) gene clusters encoding a secretion apparatus that functions like a molecular syringe. The T3SS secretes effector proteins into the target-cell cytosol, which manipulate host-cell signaling cascades. These effector proteins are suggested to have multiple activities within host cells; for example, SopB is involved in invasion and Akt activation, which causes fluid secretion and *Salmonella* containing vacuole (SCV) formation (Figure 3a) [33]–[36]. The majority of genes encoding for these virulence-associated effector molecules are located on the *S*PIs. Other effector proteins such as SopE, SspH1, SseI, SodC-1, and SopE2 are encoded by phages or phage remnants [22], [37], [38].

Of the 21 *S*PIs known to date, *S*PI-1 and *S*PI-2 are the most studied. *S.* Typhimurium and *S.* Typhi genomes share 11 common *S*PIs; four are specific to *S.* Typhi (*S*PI-7, 15, 17, and 18) and only one (*S*PI-14) for *S.* Typhimurium [20]. *S*PI-1 harbors the genes for T3SS1, which is crucial for the invasion of non-phagocytic cells such as M cells in the gut lumen and activation of pro-inflammatory responses [39]. However, *S.* Typhimurium mutants deficient in *S*PI-1 can disseminate and cause systemic infection from the gastrointestinal tract by CD-18 expressing phagocytic cells such as dendritic cells (DCs) without disrupting the epithelial barrier [40], [41]. Therefore, this alternative pathway via CD-18 immune cells facilitates the development of a systemic infection for intracellular-adapted bacteria like *Salmonella*. T3SS2, encoded on *S*PI-2, plays a crucial role during the second phase of invasion, intracellular survival in macrophages [42]. Within the phagocyte, T3SS2 prevents trafficking from the phagocyte NADPH oxidase (nicotinamide adenine dinucleotide phosphate-oxidase) towards the SCV, thereby preventing a phagocytic burst [43], [44]. Although the importance of these systems for the virulence of *S.* Typhimurium is clear, limited data are available concerning the role of T3SS in *S.* Typhi. Of note, the *S*PI-2 T3SS of *S.* Typhi is not required for survival in human macrophages but may be used during infection of other cell types, such as DCs or natural killer cells, leading to the notion that the *S*PI-2 T3SS may be required to modulate the host immune system to establish long-term asymptomatic infection [45].

Bacterial outer membrane vesicles (OMV) have been recently identified as another method used by *Salmonella* to transfer its virulence factors into the cytoplasm of the host cell [46]. For example, *S.* Typhi uses the OMV to enclose ClyA, a pore-forming cytotoxin, and subsequently release this virulence factor extracellulary [47]. Moreover, it has also been shown that OMVs can stimulate responses important for the activation of DCs, priming *Salmonella*-specific T and B cells, and possess pro-inflammatory and antigenic function, which makes them therefore attractive as vaccine candidates [48].

#### Fimbrae and flagella

Fimbriae or pili are found on the bacterial surface and are thought to be mainly important for biofilm formation, colonization, and initial attachment to the host cells, although little is known about their true virulence potential [20], [36]. Each *Salmonella* serovar harbors a unique combination of fimbrial operons. Flagella are long helical filaments attached to rotary motors embedded within the membrane that enable *Salmonella* species to travel to the epithelial barrier after ingestion. In vitro, flagellin causes upregulation of pro-inflammatory cytokines in tissue culture models [49]. However, in vivo data showed that the role of flagella in virulence can be dispensable and model-dependent [50]–[52].

#### Polysaccharides and other putative virulence factors

The polysaccharidic capsule Vi antigen is of key importance for *S.* Typhi virulence, but notably absent in *S.* Typhimurium, *S.* Paratyphi A, and most other *Salmonella* serovars. Its presence increases infectivity of *S.* Typhi and disease severity, and natural infection is usually associated with the expression of Vi antigen in isolated *S.* Typhi [53]. However, Vi negative mutants are still able to cause a typhoid-like illness in human volunteers [54]. Two widely separated chromosomal regions, *ViaA* together with *ViaB*, located on *S*PI-7, are needed for Vi synthesis [55]. Recently it has been hypothesized that the Vi capsule can prevent host-pathogen recognition by preventing lipopolysaccharide (LPS) recognition by pattern recognition receptors (PRRs) [56]. In this way *S.* Typhi does not elicit a neutrophil influx in the small bowel but is able to disseminate systemically and lead to a persistent bacterial infection [3]. Other well-documented virulence factors include ion transporters and superoxide-dismutases [36].

## The Host Defense

PRRs, most notably the Toll-like receptors (TLR) and NOD-like receptors (NLR), are the first component of the immune system to detect host invasion by pathogens, initiate immune responses, and form the crucial link between innate and adaptive immunity [57], [58]. PRRs recognize conserved motifs on pathogens termed “pathogen-associated-molecular-patterns” (PAMPs) and are also able to recognize endogenous danger signals or “danger-associated molecular-patterns” (DAMPs). During invasive *Salmonella* infection, PAMPs and DAMPs initiate the innate immune system leading to activation and recruitment of neutrophils and macrophages and the production of pro-inflammatory cytokines, most notably Interleukin (IL)-6, IL-1β, tumor necrosis factor (TNF)-α, and interferon-gamma (IFN)-γ (Figure 3b) [59]–[65]. IFN-γ plays a central role in the control of persistent infection by affecting the extent of macrophage activation [64]. IL-18 is important for IFN-γ release and early host resistance to *Salmonella* infections [66].

During severe bacterial infection uncontrolled activation of the innate immune response can lead to detrimental systemic inflammation, intravascular coagulation, tissue injury, and eventually death [67], [68]. This hyper pro-inflammatory response is only seen to a limited extent in patients with typhoid fever. Coagulation abnormalities do not become clinically apparent and serum levels of TNF-α and IL-1β are low compared to the levels measured in patients with systemic infections caused by other Gram-negative bacteria [60], [62]. Patients with typhoid fever demonstrate a distinct and highly reproducible signature in the peripheral blood, shown by micro-arrays and transcriptional profiling, that changes during treatment and convalescence, returning in the majority of cases to a normal profile as measured in healthy uninfected controls [63]. Patients who do return to a normal profile may be genetically or temporarily incapable of developing an effective immune response and may be more susceptible to re-infection, relapse, or the establishment of a carrier state [63]. In this respect it is of importance to note that antimicrobial treatment—which can lead to depletion of the gut microbiome—is associated with prolonged deleterious effects on intestinal *Salmonella* colonization resistance, which can result in increased fecal shedding and carrier status (Box 1) [69]. In contrast to NTS, which is an important cause of morbidity and mortality in patients with an inherited or acquired immunodeficiency syndrome such as HIV infection, IL-12R deficiency, or chronic granulomatous disease, typhoid fever has not been associated with any primary or acquired immunodeficiency or underlying disease [9], [70], [71]. Such a difference can potentially be ascribed to a difference in signaling through PRRs where production of interleukin-17-producing T cells and their associated family cytokines (IL-17, -21, -22, -26) play an important role in the dissemination of NTS but not *S.* Typhi [9].

**Box 1. *Salmonella* and the Gut Microbiome**

In recent years it has become clear that the intestinal microbiome, consisting of more bacteria than the total number of cells in the human body, can be seen as an exteriorised organ that exerts numerous functions in the host response against Salmonellosis. The gut hosts ∼1×10^14^ bacteria from 500–1,000 different species of which three bacterial divisions—the *Firmicutes* (Gram-positive), *Bacteroides* (Gram-negative), and *Actinobacteria* (Gram-positive)—dominate. The healthy gut microbiome can protect against epithelial cell injury by producing toxic metabolites known to repress *Salmonella* virulence gene expression, optimizes host immune systems, and mediates pathogen clearance from the gut lumen after non-typhoidal *Salmonella* diarrhea [69], [91]. Of importance, antimicrobial treatment depletes the gut microbiome and is associated with prolonged deleterious effects on intestinal *Salmonella* colonization resistance, which can result in increased fecal shedding and carrier status induction [69], [92], [93]. Ingeniously, *S.* Typhimurium is able to exploit a specific microbiome-derived nutrient, named ethanolamine, in order to acquire a significant growth advantage in the lumen of inflamed intestine over other potential pathogens [94]. Similarly, *S.* Typhimurium virulence factors have been found to induce host-driven production of a new electron acceptor that allows the pathogen to use respiration to compete with fermenting gut microbes [95]. The potential of microbiota-based therapies for curing *Salmonella*-infected patients has opened a whole new area of research [69].

### Pattern Recognition Receptors

*Salmonella* spp. expresses multiple PAMPs, most notably T3SS, flagella, fimbrae, LPS (Vi antigen), and bacterial DNA, which are recognized by PRRs (Figure 2). Not surprisingly, TLR4 plays an important role in invasive Salmonellosis. TLR4-deficient mice have increased susceptibility to *Salmonella* infection, and stimulation of TLR4 by LPS has an important role in the development of septic shock during *S.* Typhimurium infection [56], [72]–[74]. *Salmonella* flagellin leads to TLR5 activation [49]. Intriguingly, the Vi capsule expressed in mutated *S.* Typhimurium prevents both in vitro and in vivo recognition of *Salmonella* LPS by TLR4 [50], [56]. The presence of the Vi antigen on the cell surface leads to capsule formation, which ultimately prevents this recognition. A crucial role is ascribed to the TviA regulatory protein, encoded on the *S.* Typhi–specific *S*PI-7 (*viaB* locus), which downregulates flagellin production and enhances Vi antigen attachment to the cell surface [75]. The TviA protein therefore serves as a regulatory switch affecting the ability of the host to recognize *S.* Typhi as an intruder at crucial stages of the spread in humans.

The inflammasomes, which are intracellular complexes consisting of caspase-1, NLRs (e.g., NLRP3- or NLRC4), and the adaptor molecule ASC (apoptosis-associated speck-like protein containing a CARD), play a central role in the innate immune defense against *S.* Typhimurium [76]. Mice deficient in caspase-1 or the end product of inflammasome activation, namely IL-1β and IL-18, have higher bacterial load and succumb earlier upon infection with *S.* Typhimurium [77]. In vitro experiments have shown that *S.* Typhimurium induces caspase-1 and NLRC4-dependent IL-1β release and cell death [78]. NLRC4 is able to recognize bacterial flagellin that—as has been hypothesized by different authors—can be injected accidentally into the cytoplasm by the T3SS causing cytosolic perturbations [65], [79]–[81]. However, during experimental infection mice deficient in NLRC4 are able to clear *S.* Typhimurium just as efficiently as control mice, suggesting the involvement of other inflammasomes [78]. Indeed, Broz et al. demonstrated that both NLRP3 and NLRC4 activate caspase-1 in response to *S.* Typhimurium infection, and mice lacking both *NLRP3* and *NLRC4* genes show increased susceptibility to infection. In response to a bacterial trigger, NLRP3 and NLRC4 will recruit ASC and caspase-1 into a single cytoplasmic focus, which subsequently serves as the site for pro-IL-1β processing [82]. Recent work provides evidence that the recognition of bacterial flagellin by the NLRC4 inflammasome in splenic dendritic cells triggers rapid release of IL-18, which leads to IFN-γ production by memory CD8^+^ T cells [65]. NLRC4-mediated release of IL-1β has also been shown to be flagellin dependent, while the bacterial trigger for NLRP3 remains unclear. However, NLRP3 is able to recognize *S*PI-2 T3SS mutants, which lack the capacity to replicate intracellularly; therefore, the receptor is thought to play a role in the detection of persistent bacteria [83]. Although these studies underscore the central role of the inflammasome during *S.* Typhimurium infection, further research is needed to define its role in typhoid fever [83].

### Apoptosis and Pyroptosis

Apoptosis or programmed cell death is regarded as a protective mechanism of host defense by preventing further release of pro-inflammatory cellular mediators [84]. Serovars of *S. enterica* are able to employ different mechanisms to induce macrophage cell death [85], [86]. Recently it has been proposed that activation of caspase-1 can also trigger a form of pro-inflammatory cell death called “pyroptosis” [87]. Caspase-1, which is triggered via the flagellin-detection of the NRLC4-inflammasome, is able to cause clearance of *S.* Typhimurium independent of IL-1β or IL-18 by pyroptotic macrophage death [76]. In infected cells, caspase-1-induced lysis of macrophages can result in the release of bacteria into the extracellular space, which will enable efficient reactive oxygen species (ROS) mediated killing by neutrophils. During the systemic phase of infection, *S.* Typhimurium is able to completely suppress flagellin expression, which results in evasion of NLRC4 detection and subsequent pyroptosis [76]. Interestingly selected *Salmonella*-infected caspase-1-deficient macrophages do not undergo pyroptosis but display a form of delayed cell death with features of autophagy [86].

## Conclusion

Significant progress has been made in our understanding of host–pathogen interactions in invasive Salmonellosis. Mouse models using *S.* Typhimurium have been instrumental in unraveling complex pathways and have shed new light on the role of key virulence factors such as Vi antigen and T3SS (Box 2). Studies of genome differences between *S.* Typhi, *S.* Paratyphi A, *S.* Typhimurium, and other *Salmonella* serovars have begun to explain some of the variation in disease manifestations. The important yet undefined roles of DAMPs and NLR-recognition in typhoid fever remain to be clarified, and these may be major players in the severe gut ulceration that is an important cause of the mortality. Despite these major advances, large gaps remain in our understanding of the pathogenesis of the disease in humans. Unraveling of the pathogenesis of invasive Salmonellosis hopefully leads to new therapeutic treatment strategies, urgently needed in the light of growing antimicrobial resistance. Box 3 summarizes some important questions for future research on invasive *Salmonella* pathogenesis research.

**Box 2. Mouse Models for *S.* Typhi Infection**

*S.* Typhi infects humans exclusively. The consequent lack of animal models has hampered the study of host–pathogen interactions in typhoid fever. To overcome this problem experimental murine *S.* Typhimurium infection has been used extensively as a model for typhoid fever. The intestinal pathology and inflammatory response seen in this model resembles the changes observed in patients with typhoid fever [96]. Current murine models include both oral and systemic (intravenous or intraperitoneal) inoculation with or without streptomycin pretreatment [91]. Infection of susceptible mouse strains that carry a mutation in the gene encoding for a metal transporter present on the SCV membrane named *Nramp1* (*Slc11a1*), such as CL57/BL6 or BALB/C mice, produces a disease that resembles typhoid fever upon inoculation with *S.* Typhimurium [20]. To study chronic and persistent infection such as can be seen in *S.* Typhi carriers, strains of mice possessing the *Nramp* +/+ allele, which are consequently resistant to the infection with *S.* Typhimurium, are used [64]. Of note, no allelic association was identified in humans between the *Nramp* alleles and typhoid susceptibility, and as *S.* Typhimurium causes a different disease in humans than *S.* Typhi, conclusions regarding typhoid fever pathogenesis derived from animal experiments must be interpreted carefully [97]. An ingenious mouse model for *S.* Typhi was proposed by making use of immunodeficient *Rag2*−/− *yc*−/− mice engrafted with human fetal liver hematopoietic stem and progenitor cells creating humanized mice susceptible to *S.* Typhi [98]. Although these humanized mice were able to support *S.* Typhi replication and persistent infection, it did not lead to an acute lethal infection [98]. Most recently, another murine lethal *S.* Typhi model resembling characteristic features of human typhoid fever was created by making use of humanized nonobese diabetic-*scid IL2rγ^null^* mice, which are engrafted with human hematopoetic stem cells (hu-SRC-SCID mice). This model, which has already been proven to be useful for detecting new virulence determinants, could also be useful to study host–pathogen interactions and evaluate vaccine candidates [99].

**Box 3. Questions for Future Research**

**Genomics**

- Can bacterial genotype be linked to the clinical disease phenotype in humans?
- Is there a true association between bacterial genotype and/or the presence of a multidrug resistant plasmid and disease severity? If there is an association, what is the mechanism?

**Host-response**

- Is there a difference between *S.* Typhi- and *S.* Paratyphi-induced enteric fever in the host gene expression pathways?
- What is the role of DAMPs during severe typhoid fever? Are these danger-associated molecular patterns causing the damage that occurs in the gut, or are they mere bystanders triggering the NLRP3 inflammasome?
- Is a change in gut flora the reason that typhoid patients still have an altered immune profile nine months after infection? Moreover, do these patients have an increased risk for re-infection with other invasive *Salmonellae*?

**Treatment**

- Can we exploit a better understanding of disease pathogenesis to lead to new therapeutic approaches? Potential immunomodulating treatment strategies for invasive Salmonellosis could target the pathogen directly (e.g., based on drugs targeting T3SS or flagella) or target key host response proteins (e.g., TLR4, NLRC4, or IL-1β) depending on the phase of the immune response.
- Are steroids beneficial in severe typhoid fever as has previously been suggested [100]? And if so, what is the mechanism behind these observations?

## Search Strategy and Selection Criteria

Data for this review were identified by searches of PubMed, with the search terms “Typhoid Fever” in combination with “epidemiology,” “clinical features,” “therapy,” and “origin.” “Salmonella Typhi” or “Salmonella Typhimurium” in combination with “genome,” “virulence factors,” “toll-like receptors,” “NOD-like receptors,” and “inflammasome.” The references of identified articles were manually searched for further relevant papers, and we also searched our own reference databases. English and French papers were reviewed.

## References

[1] Crump JA, Mintz ED (2010) Global trends in typhoid and paratyphoid Fever. Clin Infect Dis 50: 241–246. 20014951 10.1086/649541 PMC2798017

[2] Connor BA, Schwartz E (2005) Typhoid and paratyphoid fever in travellers. Lancet Infect Dis 5: 623–628. 16183516 10.1016/S1473-3099(05)70239-5

[3] Tsolis RM, Young GM, Solnick JV, Baumler AJ (2008) From bench to bedside: stealth of enteroinvasive pathogens. Nat Rev Microbiol 6: 883–892. 18955984 10.1038/nrmicro2012

[4] Arjyal A, Basnyat B, Koirala S, Karkey A, Dongol S, et al (2011) Gatifloxacin versus chloramphenicol for uncomplicated enteric fever: an open-label, randomised, controlled trial. Lancet Infect Dis 11: 445–454. 21531174 10.1016/S1473-3099(11)70089-5 PMC3108101

[5] Beeching NJ, Parry CM (2011) Outpatient treatment of patients with enteric fever. Lancet Infect Dis 11: 419–421. 21531173 10.1016/S1473-3099(11)70119-0

[6] Monack DM, Mueller A, Falkow S (2004) Persistent bacterial infections: the interface of the pathogen and the host immune system. Nat Rev Microbiol 2: 747–765. 15372085 10.1038/nrmicro955

[7] Monack DM (2011) Salmonella persistence and transmission strategies. Curr Opin Microbiol 15: 100–107. 22137596 10.1016/j.mib.2011.10.013

[8] Parry CM, Hien TT, Dougan G, White NJ, Farrar JJ (2002) Typhoid fever. N Engl J Med 347: 1770–1782. 12456854 10.1056/NEJMra020201

[9] Feasey NA, Dougan G, Kingsley RA, Heyderman RS, Gordon MA (2012) Invasive non-typhoidal salmonella disease: an emerging and neglected tropical disease in Africa. Lancet 379: 2489–2499. 22587967 10.1016/S0140-6736(11)61752-2 PMC3402672

[10] Majowicz SE, Musto J, Scallan E, Angulo FJ, Kirk M, et al (2010) The global burden of nontyphoidal Salmonella gastroenteritis. Clin Infect Dis 50: 882–889. 20158401 10.1086/650733

[11] Graham SM (2010) Nontyphoidal salmonellosis in Africa. Curr Opin Infect Dis 23: 409–414. 20736739 10.1097/QCO.0b013e32833dd25d

[12] Parry CM, Threlfall EJ (2008) Antimicrobial resistance in typhoidal and nontyphoidal salmonellae. Curr Opin Infect Dis 21: 531–538. 18725804 10.1097/QCO.0b013e32830f453a

[13] Baker S (2011) Genomic medicine has failed the poor. Nature 478: 287. 22012349 10.1038/478287a

[14] Deng W, Liou SR, Plunkett G III, Mayhew GF, Rose DJ, et al (2003) Comparative genomics of Salmonella enterica serovar Typhi strains Ty2 and CT18. J Bacteriol 185: 2330–2337. 12644504 10.1128/JB.185.7.2330-2337.2003 PMC151493

[15] Parkhill J, Dougan G, James KD, Thomson NR, Pickard D, et al (2001) Complete genome sequence of a multiple drug resistant Salmonella enterica serovar Typhi CT18. Nature 413: 848–852. 11677608 10.1038/35101607

[16] McClelland M, Sanderson KE, Spieth J, Clifton SW, Latreille P, et al (2001) Complete genome sequence of Salmonella enterica serovar Typhimurium LT2. Nature 413: 852–856. 11677609 10.1038/35101614

[17] Baker S, Dougan G (2007) The genome of Salmonella enterica serovar Typhi. Clin Infect Dis 45 Suppl 1: S29–S33. 17582565 10.1086/518143

[18] Achtman M, Wain J, Weill FX, Nair S, Zhou Z, et al (2012) Multilocus sequence typing as a replacement for serotyping in salmonella enterica. PLoS Pathog 8: e1002776 doi:10.1371/journal.ppat.1002776. 22737074 10.1371/journal.ppat.1002776 PMC3380943

[19] Raffatellu M, Wilson RP, Winter SE, Baumler AJ (2008) Clinical pathogenesis of typhoid fever. J Infect Dev Ctries 2: 260–266. 19741286 10.3855/jidc.219

[20] Sabbagh SC, Forest CG, Lepage C, Leclerc JM, Daigle F (2010) So similar, yet so different: uncovering distinctive features in the genomes of Salmonella enterica serovars Typhimurium and Typhi. FEMS Microbiol Lett 305: 1–13. 20146749 10.1111/j.1574-6968.2010.01904.x

[21] Kingsley RA, Msefula CL, Thomson NR, Kariuki S, Holt KE, et al (2009) Epidemic multiple drug resistant Salmonella Typhimurium causing invasive disease in sub-Saharan Africa have a distinct genotype. Genome Res 19: 2279–2287. 19901036 10.1101/gr.091017.109 PMC2792184

[22] Ehrbar K, Hardt WD (2005) Bacteriophage-encoded type III effectors in Salmonella enterica subspecies 1 serovar Typhimurium. Infect Genet Evol 5: 1–9. 15567133 10.1016/j.meegid.2004.07.004

[23] Lesnick ML, Reiner NE, Fierer J, Guiney DG (2001) The Salmonella spvB virulence gene encodes an enzyme that ADP-ribosylates actin and destabilizes the cytoskeleton of eukaryotic cells. Mol Microbiol 39: 1464–1470. 11260464 10.1046/j.1365-2958.2001.02360.x

[24] Huang R, Wu S, Zhang X, Zhang Y (2005) Molecular analysis and identification of virulence gene on pR(ST98) from multi-drug resistant Salmonella typhi. Cell Mol Immunol 2: 136–140. 16191420

[25] Wu S, Li Y, Xu Y, Li Q, Chu Y, et al (2010) A Salmonella enterica serovar Typhi plasmid induces rapid and massive apoptosis in infected macrophages. Cell Mol Immunol 7: 271–278. 20473323 10.1038/cmi.2010.17 PMC4003229

[26] Baker S, Hardy J, Sanderson KE, Quail M, Goodhead I, et al (2007) A novel linear plasmid mediates flagellar variation in Salmonella Typhi. PLoS Pathog 3: e59 doi:10.1371/journal.ppat.0030059. 17500588 10.1371/journal.ppat.0030059 PMC1876496

[27] Phan MD, Kidgell C, Nair S, Holt KE, Turner AK, et al (2009) Variation in Salmonella enterica serovar typhi IncHI1 plasmids during the global spread of resistant typhoid fever. Antimicrob Agents Chemother 53: 716–727. 19015365 10.1128/AAC.00645-08 PMC2630618

[28] Phan MD, Wain J (2008) IncHI plasmids, a dynamic link between resistance and pathogenicity. J Infect Dev Ctries 2: 272–278. 19741288 10.3855/jidc.221

[29] Holt KE, Phan MD, Baker S, Duy PT, Nga TV, et al (2011) Emergence of a globally dominant IncHI1 plasmid type associated with multiple drug resistant typhoid. PLoS Negl Trop Dis 5: e1245 doi:10.1371/journal.pntd.0001245. 21811646 10.1371/journal.pntd.0001245 PMC3139670

[30] Bhutta ZA (1996) Impact of age and drug resistance on mortality in typhoid fever. Arch Dis Child 75: 214–217. 8976660 10.1136/adc.75.3.214 PMC1511710

[31] Wain J, Diep TS, Ho VA, Walsh AM, Nguyen TT, et al (1998) Quantitation of bacteria in blood of typhoid fever patients and relationship between counts and clinical features, transmissibility, and antibiotic resistance. J Clin Microbiol 36: 1683–1687. 9620400 10.1128/jcm.36.6.1683-1687.1998 PMC104900

[32] Thomson N, Baker S, Pickard D, Fookes M, Anjum M, et al (2004) The role of prophage-like elements in the diversity of Salmonella enterica serovars. J Mol Biol 339: 279–300. 15136033 10.1016/j.jmb.2004.03.058

[33] Terebiznik MR, Vieira OV, Marcus SL, Slade A, Yip CM, et al (2002) Elimination of host cell PtdIns(4,5)P(2) by bacterial SigD promotes membrane fission during invasion by Salmonella. Nat Cell Biol 4: 766–773. 12360287 10.1038/ncb854

[34] Hernandez LD, Hueffer K, Wenk MR, Galan JE (2004) Salmonella modulates vesicular traffic by altering phosphoinositide metabolism. Science 304: 1805–1807. 15205533 10.1126/science.1098188

[35] Knodler LA, Finlay BB, Steele-Mortimer O (2005) The Salmonella effector protein SopB protects epithelial cells from apoptosis by sustained activation of Akt. J Biol Chem 280: 9058–9064. 15642738 10.1074/jbc.M412588200

[36] Ibarra JA, Steele-Mortimer O (2009) Salmonella—the ultimate insider. Salmonella virulence factors that modulate intracellular survival. Cell Microbiol 11: 1579–1586. 19775254 10.1111/j.1462-5822.2009.01368.x PMC2774479

[37] Fang FC, DeGroote MA, Foster JW, Baumler AJ, Ochsner U, et al (1999) Virulent Salmonella typhimurium has two periplasmic Cu, Zn-superoxide dismutases. Proc Natl Acad Sci U S A 96: 7502–7507. 10377444 10.1073/pnas.96.13.7502 PMC22115

[38] Figueroa-Bossi N, Bossi L (1999) Inducible prophages contribute to Salmonella virulence in mice. Mol Microbiol 33: 167–176. 10411733 10.1046/j.1365-2958.1999.01461.x

[39] Galan JE (1999) Interaction of Salmonella with host cells through the centisome 63 type III secretion system. Curr Opin Microbiol 2: 46–50. 10047557 10.1016/s1369-5274(99)80008-3

[40] Vazquez-Torres A, Jones-Carson J, Baumler AJ, Falkow S, Valdivia R, et al (1999) Extraintestinal dissemination of Salmonella by CD18-expressing phagocytes. Nature 401: 804–808. 10548107 10.1038/44593

[41] Rescigno M, Urbano M, Valzasina B, Francolini M, Rotta G, et al (2001) Dendritic cells express tight junction proteins and penetrate gut epithelial monolayers to sample bacteria. Nat Immunol 2: 361–367. 11276208 10.1038/86373

[42] Hensel M, Shea JE, Waterman SR, Mundy R, Nikolaus T, et al (1998) Genes encoding putative effector proteins of the type III secretion system of Salmonella pathogenicity island 2 are required for bacterial virulence and proliferation in macrophages. Mol Microbiol 30: 163–174. 9786193 10.1046/j.1365-2958.1998.01047.x

[43] Vazquez-Torres A, Xu Y, Jones-Carson J, Holden DW, Lucia SM, et al (2000) Salmonella pathogenicity island 2-dependent evasion of the phagocyte NADPH oxidase. Science 287: 1655–1658. 10698741 10.1126/science.287.5458.1655

[44] Gallois A, Klein JR, Allen LA, Jones BD, Nauseef WM (2001) Salmonella pathogenicity island 2-encoded type III secretion system mediates exclusion of NADPH oxidase assembly from the phagosomal membrane. J Immunol 166: 5741–5748. 11313417 10.4049/jimmunol.166.9.5741

[45] Forest CG, Ferraro E, Sabbagh SC, Daigle F (2010) Intracellular survival of Salmonella enterica serovar Typhi in human macrophages is independent of Salmonella pathogenicity island (SPI)-2. Microbiology 156: 3689–3698. 20817644 10.1099/mic.0.041624-0

[46] Yoon H, Ansong C, Adkins JN, Heffron F (2011) Discovery of Salmonella virulence factors translocated via outer membrane vesicles to murine macrophages. Infect Immun 79: 2182–2192. 21464085 10.1128/IAI.01277-10 PMC3125828

[47] Wai SN, Lindmark B, Soderblom T, Takade A, Westermark M, et al (2003) Vesicle-mediated export and assembly of pore-forming oligomers of the enterobacterial ClyA cytotoxin. Cell 115: 25–35. 14532000 10.1016/s0092-8674(03)00754-2

[48] Alaniz RC, Deatherage BL, Lara JC, Cookson BT (2007) Membrane vesicles are immunogenic facsimiles of Salmonella typhimurium that potently activate dendritic cells, prime B and T cell responses, and stimulate protective immunity in vivo. J Immunol 179: 7692–7701. 18025215 10.4049/jimmunol.179.11.7692

[49] Zeng H, Carlson AQ, Guo Y, Yu Y, Collier-Hyams LS, et al (2003) Flagellin is the major proinflammatory determinant of enteropathogenic Salmonella. J Immunol 171: 3668–3674. 14500664 10.4049/jimmunol.171.7.3668

[50] Winter SE, Thiennimitr P, Nuccio SP, Haneda T, Winter MG, et al (2009) Contribution of flagellin pattern recognition to intestinal inflammation during Salmonella enterica serotype typhimurium infection. Infect Immun 77: 1904–1916. 19237529 10.1128/IAI.01341-08 PMC2681779

[51] Lockman HA, Curtiss R 3rd (1990) Salmonella typhimurium mutants lacking flagella or motility remain virulent in BALB/c mice. Infect Immun 58: 137–143. 2152887 10.1128/iai.58.1.137-143.1990 PMC258421

[52] Schmitt CK, Ikeda JS, Darnell SC, Watson PR, Bispham J, et al (2001) Absence of all components of the flagellar export and synthesis machinery differentially alters virulence of Salmonella enterica serovar Typhimurium in models of typhoid fever, survival in macrophages, tissue culture invasiveness, and calf enterocolitis. Infect Immun 69: 5619–5625. 11500437 10.1128/IAI.69.9.5619-5625.2001 PMC98677

[53] Wain J, House D, Zafar A, Baker S, Nair S, et al (2005) Vi antigen expression in Salmonella enterica serovar Typhi clinical isolates from Pakistan. J Clin Microbiol 43: 1158–1165. 15750077 10.1128/JCM.43.3.1158-1165.2005 PMC1081282

[54] Zhang XL, Jeza VT, Pan Q (2008) Salmonella typhi: from a human pathogen to a vaccine vector. Cell Mol Immunol 5: 91–97. 18445338 10.1038/cmi.2008.11 PMC4651240

[55] Kolyva S, Waxin H, Popoff MY (1992) The Vi antigen of Salmonella typhi: molecular analysis of the viaB locus. J Gen Microbiol 138: 297–304. 1564441 10.1099/00221287-138-2-297

[56] Wilson RP, Raffatellu M, Chessa D, Winter SE, Tukel C, et al (2008) The Vi-capsule prevents Toll-like receptor 4 recognition of Salmonella. Cell Microbiol 10: 876–890. 18034866 10.1111/j.1462-5822.2007.01090.x

[57] Kawai T, Akira S (2010) The role of pattern-recognition receptors in innate immunity: update on Toll-like receptors. Nat Immunol 11: 373–384. 20404851 10.1038/ni.1863

[58] Schroder K, Tschopp J (2010) The inflammasomes. Cell 140: 821–832. 20303873 10.1016/j.cell.2010.01.040

[59] Butler T, Ho M, Acharya G, Tiwari M, Gallati H (1993) Interleukin-6, gamma interferon, and tumor necrosis factor receptors in typhoid fever related to outcome of antimicrobial therapy. Antimicrob Agents Chemother 37: 2418–2421. 8285627 10.1128/aac.37.11.2418 PMC192401

[60] Butler T, Bell WR, Levin J, Linh NN, Arnold K (2011) Typhoid fever. Studies of blood coagulation, bacteremia, and endotoxemia. Arch Intern Med 138: 407–410. 10.1001/archinte.138.3.407 629635

[61] Keuter M, Dharmana E, Gasem MH, van dV, Djokomoeljanto R, et al (1994) Patterns of proinflammatory cytokines and inhibitors during typhoid fever. J Infect Dis 169: 1306–1311. 8195608 10.1093/infdis/169.6.1306

[62] Raffatellu M, Chessa D, Wilson RP, Tukel C, Akcelik M, et al (2006) Capsule-mediated immune evasion: a new hypothesis explaining aspects of typhoid fever pathogenesis. Infect Immun 74: 19–27. 16368953 10.1128/IAI.74.1.19-27.2006 PMC1346610

[63] Thompson LJ, Dunstan SJ, Dolecek C, Perkins T, House D, et al (2009) Transcriptional response in the peripheral blood of patients infected with Salmonella enterica serovar Typhi. Proc Natl Acad Sci U S A 106: 22433–22438. 20018727 10.1073/pnas.0912386106 PMC2792164

[64] Monack DM, Bouley DM, Falkow S (2004) Salmonella typhimurium persists within macrophages in the mesenteric lymph nodes of chronically infected Nramp1+/+ mice and can be reactivated by IFNgamma neutralization. J Exp Med 199: 231–241. 14734525 10.1084/jem.20031319 PMC2211772

[65] Kupz A, Guarda G, Gebhardt T, Sander LE, Short KR, et al (2012) NLRC4 inflammasomes in dendritic cells regulate noncognate effector function by memory CD8(+) T cells. Nat Immunol 13: 162–169. 22231517 10.1038/ni.2195

[66] Mastroeni P, Clare S, Khan S, Harrison JA, Hormaeche CE, et al (1999) Interleukin 18 contributes to host resistance and gamma interferon production in mice infected with virulent Salmonella typhimurium. Infect Immun 67: 478–483. 9916048 10.1128/iai.67.2.478-483.1999 PMC96344

[67] de Jong HK, van der Poll T, Wiersinga WJ (2010) The systemic pro-inflammatory response in sepsis. J Innate Immun 2: 422–430. 20530955 10.1159/000316286

[68] van der Poll T, Opal SM (2008) Host-pathogen interactions in sepsis. Lancet Infect Dis 8: 32–43. 18063412 10.1016/S1473-3099(07)70265-7

[69] Endt K, Stecher B, Chaffron S, Slack E, Tchitchek N, et al (2010) The microbiota mediates pathogen clearance from the gut lumen after non-typhoidal Salmonella diarrhea. PLoS Pathog 6: e1001097 doi:10.1371/journal.ppat.1001097. 20844578 10.1371/journal.ppat.1001097 PMC2936549

[70] Raffatellu M, Santos RL, Verhoeven DE, George MD, Wilson RP, et al (2008) Simian immunodeficiency virus-induced mucosal interleukin-17 deficiency promotes Salmonella dissemination from the gut. Nat Med 14: 421–428. 18376406 10.1038/nm1743 PMC2901863

[71] Gordon MA, Kankwatira AM, Mwafulirwa G, Walsh AL, Hopkins MJ, et al (2010) Invasive non-typhoid salmonellae establish systemic intracellular infection in HIV-infected adults: an emerging disease pathogenesis. Clin Infect Dis 50: 953–962. 20180702 10.1086/651080

[72] O'Brien AD, Rosenstreich DL, Taylor BA (1980) Control of natural resistance to Salmonella typhimurium and Leishmania donovani in mice by closely linked but distinct genetic loci. Nature 287: 440–442. 7001248 10.1038/287440a0

[73] Weinstein DL, Lissner CR, Swanson RN, O'Brien AD (1986) Macrophage defect and inflammatory cell recruitment dysfunction in Salmonella susceptible C3H/HeJ mice. Cell Immunol 102: 68–77. 3542230 10.1016/0008-8749(86)90326-6

[74] Vazquez-Torres A, Vallance BA, Bergman MA, Finlay BB, Cookson BT, et al (2004) Toll-like receptor 4 dependence of innate and adaptive immunity to Salmonella: importance of the Kupffer cell network. J Immunol 172: 6202–6208. 15128808 10.4049/jimmunol.172.10.6202

[75] Winter SE, Raffatellu M, Wilson RP, Russmann H, Baumler AJ (2008) The Salmonella enterica serotype Typhi regulator TviA reduces interleukin-8 production in intestinal epithelial cells by repressing flagellin secretion. Cell Microbiol 10: 247–261. 17725646 10.1111/j.1462-5822.2007.01037.x

[76] Miao EA, Leaf IA, Treuting PM, Mao DP, Dors M, et al (2010) Caspase-1-induced pyroptosis is an innate immune effector mechanism against intracellular bacteria. Nat Immunol 11: 1136–1142. 21057511 10.1038/ni.1960 PMC3058225

[77] Raupach B, Peuschel SK, Monack DM, Zychlinsky A (2006) Caspase-1-mediated activation of interleukin-1beta (IL-1beta) and IL-18 contributes to innate immune defenses against Salmonella enterica serovar Typhimurium infection. Infect Immun 74: 4922–4926. 16861683 10.1128/IAI.00417-06 PMC1539628

[78] Lara-Tejero M, Sutterwala FS, Ogura Y, Grant EP, Bertin J, et al (2006) Role of the caspase-1 inflammasome in Salmonella typhimurium pathogenesis. J Exp Med 203: 1407–1412. 16717117 10.1084/jem.20060206 PMC2118315

[79] Franchi L, Amer A, Body-Malapel M, Kanneganti TD, Ozoren N, et al (2006) Cytosolic flagellin requires Ipaf for activation of caspase-1 and interleukin 1beta in salmonella-infected macrophages. Nat Immunol 7: 576–582. 16648852 10.1038/ni1346

[80] Miao EA, Alpuche-Aranda CM, Dors M, Clark AE, Bader MW, et al (2006) Cytoplasmic flagellin activates caspase-1 and secretion of interleukin 1beta via Ipaf. Nat Immunol 7: 569–575. 16648853 10.1038/ni1344

[81] Miao EA, Mao DP, Yudkovsky N, Bonneau R, Lorang CG, et al (2010) Innate immune detection of the type III secretion apparatus through the NLRC4 inflammasome. Proc Natl Acad Sci U S A 107: 3076–3080. 20133635 10.1073/pnas.0913087107 PMC2840275

[82] Broz P, Newton K, Lamkanfi M, Mariathasan S, Dixit VM, et al (2010) Redundant roles for inflammasome receptors NLRP3 and NLRC4 in host defense against Salmonella. J Exp Med 207: 1745–1755. 20603313 10.1084/jem.20100257 PMC2916133

[83] Broz P, Monack DM (2011) Molecular mechanisms of inflammasome activation during microbial infections. Immunol Rev 243: 174–190. 21884176 10.1111/j.1600-065X.2011.01041.x PMC3170129

[84] Fink SL, Cookson BT (2005) Apoptosis, pyroptosis, and necrosis: mechanistic description of dead and dying eukaryotic cells. Infect Immun 73: 1907–1916. 15784530 10.1128/IAI.73.4.1907-1916.2005 PMC1087413

[85] Hueffer K, Galan JE (2004) Salmonella-induced macrophage death: multiple mechanisms, different outcomes. Cell Microbiol 6: 1019–1025. 15469431 10.1111/j.1462-5822.2004.00451.x

[86] Hernandez LD, Pypaert M, Flavell RA, Galan JE (2003) A Salmonella protein causes macrophage cell death by inducing autophagy. J Cell Biol 163: 1123–1131. 14662750 10.1083/jcb.200309161 PMC2173598

[87] Brodsky IE, Monack D (2009) NLR-mediated control of inflammasome assembly in the host response against bacterial pathogens. Semin Immunol 21: 199–207. 19539499 10.1016/j.smim.2009.05.007

[88] Crawford RW, Rosales-Reyes R, Ramirez-Aguilar MdeL, Chapa-Azuela O, Alpuche-Aranda C, et al (2010) Gallstones play a significant role in Salmonella spp. gallbladder colonization and carriage. Proc Natl Acad Sci U S A 107: 4353–4358. 20176950 10.1073/pnas.1000862107 PMC2840110

[89] Everest P, Wain J, Roberts M, Rook G, Dougan G (2001) The molecular mechanisms of severe typhoid fever. Trends Microbiol 9: 316–320. 11435104 10.1016/s0966-842x(01)02067-4

[90] Kobayashi K, Hernandez LD, Galan JE, Janeway CA Jr, Medzhitov R, et al (2002) IRAK-M is a negative regulator of Toll-like receptor signaling. Cell 110: 191–202. 12150927 10.1016/s0092-8674(02)00827-9

[91] Kaiser P, Diard M, Stecher B, Hardt WD (2012) The streptomycin mouse model for Salmonella diarrhea: functional analysis of the microbiota, the pathogen's virulence factors, and the host's mucosal immune response. Immunol Rev 245: 56–83. 22168414 10.1111/j.1600-065X.2011.01070.x

[92] Croswell A, Amir E, Teggatz P, Barman M, Salzman NH (2009) Prolonged impact of antibiotics on intestinal microbial ecology and susceptibility to enteric Salmonella infection. Infect Immun 77: 2741–2753. 19380465 10.1128/IAI.00006-09 PMC2708550

[93] Gopinath S, Carden S, Monack D (2012) Shedding light on Salmonella carriers. Trends Microbiol 20: 320–327. 22591832 10.1016/j.tim.2012.04.004

[94] Thiennimitr P, Winter SE, Baumler AJ (2012) Salmonella, the host and its microbiota. Curr Opin Immunol 15: 108–114. 10.1016/j.mib.2011.10.002 PMC3265626 22030447

[95] Winter SE, Thiennimitr P, Winter MG, Butler BP, Huseby DL, et al (2010) Gut inflammation provides a respiratory electron acceptor for Salmonella. Nature 467: 426–429. 20864996 10.1038/nature09415 PMC2946174

[96] Santos RL, Zhang S, Tsolis RM, Kingsley RA, Adams LG, et al (2001) Animal models of Salmonella infections: enteritis versus typhoid fever. Microbes Infect 3: 1335–1344. 11755423 10.1016/s1286-4579(01)01495-2

[97] Dunstan SJ, Ho VA, Duc CM, Lanh MN, Phuong CX, et al (2001) Typhoid fever and genetic polymorphisms at the natural resistance-associated macrophage protein 1. J Infect Dis 183: 1156–1160. 11237848 10.1086/319289 PMC2413323

[98] Song J, Willinger T, Rongvaux A, Eynon EE, Stevens S, et al (2010) A mouse model for the human pathogen Salmonella typhi. Cell Host Microbe 8: 369–376. 20951970 10.1016/j.chom.2010.09.003 PMC2972545

[99] Libby SJ, Brehm MA, Greiner DL, Shultz LD, McClelland M, et al (2010) Humanized nonobese diabetic-scid IL2rgammanull mice are susceptible to lethal Salmonella Typhi infection. Proc Natl Acad Sci U S A 107: 15589–15594. 20713716 10.1073/pnas.1005566107 PMC2932584

[100] Hoffman SL, Punjabi NH, Kumala S, Moechtar MA, Pulungsih SP, et al (1984) Reduction of mortality in chloramphenicol-treated severe typhoid fever by high-dose dexamethasone. N Engl J Med 310: 82–88. 6361558 10.1056/NEJM198401123100203

